# What the HEC happens around the heart during COVID-19?

**DOI:** 10.1007/s00395-021-00883-7

**Published:** 2021-07-05

**Authors:** Mirko Trilling

**Affiliations:** Institute for Virology, University Hospital Essen, University of Duisburg-Essen, 45147 Essen, NRW Germany

The *severe acute respiratory syndrome coronavirus 2* (SARS-CoV-2) emerged by the end of 2019 [28] and is currently causing a global pandemic. Amid the third wave of the pandemic, over 174 million laboratory-confirmed infections have occurred to date and more than 3.74 million people died in the context of a *coronavirus infectious disease 2019* (COVID-19). Projections regarding undertesting, underreporting, and excess mortalities indicate that the true numbers of cases and fatalities are actually far higher. Despite the encouraging availability of very safe and effective vaccines, it will take a long time to overcome the medical, social, and economic consequences of the COVID-19 pandemic—especially in developing countries that struggle to provide their populations with sufficient vaccines.

SARS-CoV-2 is a betacoronavirus belonging to the sarbecovirus subgenus and shares several virological as well as clinical aspects with the first *severe acute respiratory syndrome virus* that had caused a restricted epidemic in 2003 [13]. However, a distinctive feature of SARS-CoV-2 is its very efficient human-to-human transmission [23]. The most prominent disease manifestations caused by SARS-CoV-2 involve the respiratory tract. Usually, virus replication causes less severe symptoms when confined to the upper respiratory tract, whereas extensive virus replication in the lower respiratory tract can cause pneumonia, acute respiratory distress syndrome, and eventually death, especially in the elderly and individuals with pre-existing conditions. However, the SARS-CoV-2 tropisms and the organ manifestations of COVID-19 are far more complex: in addition to the respiratory tract, several other organs and tissues are also permissive for SARS-CoV-2 replication, resulting in a variety of different types of COVID-19 [8, 17]. SARS-CoV-2 is a cytopathic virus. Accordingly, its replication itself destroys host cells and impairs the functions of affected tissues. Unfortunately, critically ill patients have been found to suffer from severe COVID-19 at times when very little if any virus progeny was detectable in clinical swab samples and various body fluids. One reason is that the vigorous immune response stimulated by SARS-CoV-2 contributes to the pathophysiology of organ failure, e.g., through hyper-inflammation [19]. Accordingly, patients in later stages of disease benefit clinically from anti-inflammatory drugs such as Dexamethasone [7]. Even after the acute infection has resolved, a significant proportion of patients, rather than recovering to their pre-COVID-19 health status, continue to suffer from various forms of long-COVID syndrome [3, 15]. In terms of aforementioned organ manifestations beyond the respiratory tract and the pathologic consequences of auto-destructive inflammation as well as long-COVID, a central question is if SARS-CoV-2 can bug out in certain niches, in which the virus or at least virus-encoded antigens such as the spike (S) protein persist.

Besides aforementioned other clinical features, SARS-CoV-2 causes a spectrum of cardiac and cardiovascular manifestations [10, 14, 20]. A study using cardiac magnetic resonance imaging showed an astonishing incidence of cardiac involvement in 78% and ongoing myocardial inflammation in 60% of patients who recently recovered from COVID-19 [18]. Upon SARS-CoV-2 infection, people with pre-existing cardiovascular diseases are at an increased risk of severe disease and death, and the coronavirus infection is associated with multiple cardiovascular complications including acute myocardial injury, myocarditis, arrhythmias, and venous thromboembolism [4]. Given that the most relevant entry receptor of SARS-CoV-2, the *human angiotensin-converting enzyme 2* (hACE2) [9], is expressed in the endothelium of arteries and veins, COVID-19 has been described to be almost as much a vascular disease as it is a respiratory disease [2, 12].

In these regards, Stefanie Dimmeler and colleagues performed a comprehensive characterization of the SARS-CoV-2 permissiveness of various human endothelial cells (HEC) derived from different vascular beds. Whereas the expression of the protease TMPRSS2 was not detectable in any of the tested endothelial cells, only *coronary artery endothelial cells* (HCAEC) were found to express hACE2—albeit with an unusual intracellular localization, largely restricted to intracellular compartments, but not on the cell surface. Consistent with the hACE2 expression, only HCAEC tested positive for the viral S protein upon exposure to SARS-CoV-2. Intriguingly, infections with the newly emerging *variants of concern* (VOC) such as B.1.1.7, B.1.351, and P.2, first identified in UK, South Africa and Brazil, respectively, resulted in significantly elevated S levels in HCAEC. Although SARS-CoV-2 enters HCAEC as indicated by the reproducible, significant, and dose-dependent intracellular presence of the S protein, neither the characteristic double-stranded RNA intermediates occurring during viral genome amplification nor infectious progeny virus in the cell supernatant were detected, indicating that SARS-CoV-2 is capable to enter but incapable to replicate in HCAEC, providing compelling evidence for an abortive type of infection of human coronary artery endothelial cells by SARS-CoV-2.

Like all relevant research, this well-conducted study answers several important questions while also raising a number of intriguing new ones: how frequent and under which circumstances do SARS-CoV-2, and VOCs in particular, reach HCAECs *in natura*? To which extent do abortive HCAEC infections contribute to the known cardiovascular manifestations of COVID-19 [1, 5]? Which post-entry mechanisms prevent productive SARS-CoV-2 replication in HCAECs? Does the S protein present in HCAEC result in immune responses mediated by described S-specific lymphocytes [6, 11, 21] or antibodies [24–26]?

Taken together, the study by Dimmeler and colleagues published in this issue of *Basic Research in Cardiology* [22], in line with concordant research conducted by others in iPSC models [16, 27], uncovers a remarkable aspect of the biology and the pathophysiology of COVID-19 by showing that SARS-CoV-2 is capable to cause abortive infections of human coronary artery endothelial cells.

## References

[1] Akhmerov A, Marban E (2020). COVID-19 and the Heart. Circ Res.

[2] Bonow RO, O'Gara PT, Yancy CW (2020). Cardiology and COVID-19. JAMA.

[3] Carfi A, Bernabei R, Landi F, Gemelli Against C-P-ACSG (2020). Persistent symptoms in patients after acute COVID-19. JAMA.

[4] Driggin E, Madhavan MV, Bikdeli B, Chuich T, Laracy J, Biondi-Zoccai G, Brown TS, Der Nigoghossian C, Zidar DA, Haythe J, Brodie D, Beckman JA, Kirtane AJ, Stone GW, Krumholz HM, Parikh SA (2020). Cardiovascular considerations for patients, health care workers, and health systems during the COVID-19 pandemic. J Am Coll Cardiol.

[5] Gori T, Lelieveld J, Munzel T (2020). Perspective: cardiovascular disease and the Covid-19 pandemic. Basic Res Cardiol.

[6] Grifoni A, Weiskopf D, Ramirez SI, Mateus J, Dan JM, Moderbacher CR, Rawlings SA, Sutherland A, Premkumar L, Jadi RS, Marrama D, de Silva AM, Frazier A, Carlin AF, Greenbaum JA, Peters B, Krammer F, Smith DM, Crotty S, Sette A (2020). Targets of T cell responses to SARS-CoV-2 coronavirus in humans with COVID-19 disease and unexposed individuals. Cell.

[7] Group RC, Horby P, Lim WS, Emberson JR, Mafham M, Bell JL, Linsell L, Staplin N, Brightling C, Ustianowski A, Elmahi E, Prudon B, Green C, Felton T, Chadwick D, Rege K, Fegan C, Chappell LC, Faust SN, Jaki T, Jeffery K, Montgomery A, Rowan K, Juszczak E, Baillie JK, Haynes R, Landray MJ (2021). Dexamethasone in hospitalized patients with Covid-19. N Engl J Med.

[8] Gupta A, Madhavan MV, Sehgal K, Nair N, Mahajan S, Sehrawat TS, Bikdeli B, Ahluwalia N, Ausiello JC, Wan EY, Freedberg DE, Kirtane AJ, Parikh SA, Maurer MS, Nordvig AS, Accili D, Bathon JM, Mohan S, Bauer KA, Leon MB, Krumholz HM, Uriel N, Mehra MR, Elkind MSV, Stone GW, Schwartz A, Ho DD, Bilezikian JP, Landry DW (2020). Extrapulmonary manifestations of COVID-19. Nat Med.

[9] Hoffmann M, Kleine-Weber H, Schroeder S, Kruger N, Herrler T, Erichsen S, Schiergens TS, Herrler G, Wu NH, Nitsche A, Muller MA, Drosten C, Pohlmann S (2020). SARS-CoV-2 cell entry depends on ACE2 and TMPRSS2 and is blocked by a clinically proven protease inhibitor. Cell.

[10] Huang C, Wang Y, Li X, Ren L, Zhao J, Hu Y, Zhang L, Fan G, Xu J, Gu X, Cheng Z, Yu T, Xia J, Wei Y, Wu W, Xie X, Yin W, Li H, Liu M, Xiao Y, Gao H, Guo L, Xie J, Wang G, Jiang R, Gao Z, Jin Q, Wang J, Cao B (2020). Clinical features of patients infected with 2019 novel coronavirus in Wuhan, China. Lancet.

[11] Li Z, Liu J, Deng H, Yang X, Wang H, Feng X, Zelinskyy G, Trilling M, Sutter K, Lu M, Dittmer U, Wang B, Yang D, Zheng X, Liu J (2020). SARS-CoV-2-specific T cell memory is long-lasting in the majority of convalsecent COVID-19 individuals. bioRxiv.

[12] Libby P, Luscher T (2020). COVID-19 is, in the end, an endothelial disease. Eur Heart J.

[13] Liu J, Zheng X, Tong Q, Li W, Wang B, Sutter K, Trilling M, Lu M, Dittmer U, Yang D (2020). Overlapping and discrete aspects of the pathology and pathogenesis of the emerging human pathogenic coronaviruses SARS-CoV, MERS-CoV, and 2019-nCoV. J Med Virol.

[14] Long B, Brady WJ, Koyfman A, Gottlieb M (2020). Cardiovascular complications in COVID-19. Am J Emerg Med.

[15] Mahase E (2020). Covid-19: what do we know about “long covid”?. BMJ.

[16] Perez-Bermejo JA, Kang S, Rockwood SJ, Simoneau CR, Joy DA, Silva AC, Ramadoss GN, Flanigan WR, Fozouni P, Li H, Chen PY, Nakamura K, Whitman JD, Hanson PJ, McManus BM, Ott M, Conklin BR, McDevitt TC (2021). SARS-CoV-2 infection of human iPSC-derived cardiac cells reflects cytopathic features in hearts of patients with COVID-19. Sci Transl Med.

[17] Puelles VG, Lutgehetmann M, Lindenmeyer MT, Sperhake JP, Wong MN, Allweiss L, Chilla S, Heinemann A, Wanner N, Liu S, Braun F, Lu S, Pfefferle S, Schroder AS, Edler C, Gross O, Glatzel M, Wichmann D, Wiech T, Kluge S, Pueschel K, Aepfelbacher M, Huber TB (2020). Multiorgan and renal tropism of SARS-CoV-2. N Engl J Med.

[18] Puntmann VO, Carerj ML, Wieters I, Fahim M, Arendt C, Hoffmann J, Shchendrygina A, Escher F, Vasa-Nicotera M, Zeiher AM, Vehreschild M, Nagel E (2020). Outcomes of cardiovascular magnetic resonance imaging in patients recently recovered from coronavirus disease 2019 (COVID-19). JAMA Cardiol.

[19] Rizzo P, Vieceli Dalla Sega F, Fortini F, Marracino L, Rapezzi C, Ferrari R (2020). COVID-19 in the heart and the lungs: could we “Notch” the inflammatory storm?. Basic Res Cardiol.

[20] Szekely Y, Lichter Y, Taieb P, Banai A, Hochstadt A, Merdler I, Gal Oz A, Rothschild E, Baruch G, Peri Y, Arbel Y, Topilsky Y (2020). Spectrum of cardiac manifestations in COVID-19: a systematic echocardiographic study. Circulation.

[21] Thieme CJ, Anft M, Paniskaki K, Blazquez-Navarro A, Doevelaar A, Seibert FS, Hoelzer B, Konik MJ, Berger MM, Brenner T, Tempfer C, Watzl C, Meister TL, Pfaender S, Steinmann E, Dolff S, Dittmer U, Westhoff TH, Witzke O, Stervbo U, Roch T, Babel N (2020). Robust T cell response toward spike, membrane, and nucleocapsid SARS-CoV-2 proteins is not associated with recovery in critical COVID-19 patients. Cell Rep Med.

[22] Wagner JUG, Bojkova D, Shumliakivska M, Luxán G, Nicin L, Aslan GS, Milting H, Kandler JD, Dendorfer A, Heumueller AW, Fleming I, Bibli S-I, Jakobi T, Dieterich C, Zeiher AM, Ciesek S, Cinatl J, Dimmeler S (2021) Increased susceptibility of human endothelial cells to infections by SARS-CoV-2 variants. Basic Res Cardiol. 10.1007/s00395-021-00882-8 10.1007/s00395-021-00882-8 PMC8256413 34224022

[23] Wang S, Trilling M, Sutter K, Dittmer U, Lu M, Zheng X, Yang D, Liu J (2020). A crowned Killer’s resume: genome, structure, receptors, and origin of SARS-CoV-2. Virol Sin.

[24] Wu J, Liang B-Y, Fang Y-H, Wang H, Yang X-L, Shen S, Chen L-K, Li S-M, Lu S-H, Xiang T-D, Liu J, Le-Trilling VTK, Lu M-J, Yang D-L, Deng F, Dittmer U, Trilling M, Zheng X (2021). Occurrence of COVID-19 symptoms during SARS-CoV-2 infection defines waning of humoral immunity. bioRxiv.

[25] Wu J, Liang B, Chen C, Wang H, Fang Y, Shen S, Yang X, Wang B, Chen L, Chen Q, Wu Y, Liu J, Yang X, Li W, Zhu B, Zhou W, Wang H, Li S, Lu S, Liu D, Li H, Krawczyk A, Lu M, Yang D, Deng F, Dittmer U, Trilling M, Zheng X (2021). SARS-CoV-2 infection induces sustained humoral immune responses in convalescent patients following symptomatic COVID-19. Nat Commun.

[26] Xiang T, Liang B, Fang Y, Lu S, Li S, Wang H, Li H, Yang X, Shen S, Zhu B, Wang B, Wu J, Liu J, Lu M, Yang D, Dittmer U, Trilling M, Deng F, Zheng X (2021). Declining levels of neutralizing antibodies against SARS-CoV-2 in convalescent COVID-19 patients one year post symptom onset. Front Immunol.

[27] Yang L, Han Y, Nilsson-Payant BE, Gupta V, Wang P, Duan X, Tang X, Zhu J, Zhao Z, Jaffre F, Zhang T, Kim TW, Harschnitz O, Redmond D, Houghton S, Liu C, Naji A, Ciceri G, Guttikonda S, Bram Y, Nguyen DT, Cioffi M, Chandar V, Hoagland DA, Huang Y, Xiang J, Wang H, Lyden D, Borczuk A, Chen HJ, Studer L, Pan FC, Ho DD, tenOever BR, Evans T, Schwartz RE, Chen S (2020). A human pluripotent stem cell-based platform to study SARS-CoV-2 tropism and model virus infection in human cells and organoids. Cell Stem Cell.

[28] Zhu N, Zhang D, Wang W, Li X, Yang B, Song J, Zhao X, Huang B, Shi W, Lu R, Niu P, Zhan F, Ma X, Wang D, Xu W, Wu G, Gao GF, Tan W, China Novel Coronavirus I, Research T (2020). A novel coronavirus from patients with pneumonia in China, 2019. N Engl J Med.

